# Higher cerebrospinal fluid biomarkers of neuronal injury in HIV-associated neurocognitive impairment

**DOI:** 10.1007/s13365-022-01081-4

**Published:** 2022-06-08

**Authors:** Ronald J. Ellis, Ahmed Chenna, Christos J. Petropoulos, Yolanda Lie, Dusica Curanovic, Melanie Crescini, John Winslow, Erin Sundermann, Bin Tang, Scott L. Letendre

**Affiliations:** 1 Departments of Neurosciences and Psychiatry, University of California, San Diego, USA; 2 Monogram Biosciences, South San Francisco, CA USA; 3 Department of Psychiatry, University of California, San Diego, USA; 4 Departments of Medicine and Psychiatry, University of California, San Diego, USA

**Keywords:** HIV, Cognition, Biomarkers, Neurodegeneration, Cerebrospinal fluid

## Abstract

We evaluated whether biomarkers of age-related neuronal injury and amyloid metabolism are associated with neurocognitive impairment (NCI) in people with and without HIV (PWH, PWoH). This was a cross-sectional study of virally suppressed PWH and PWoH. NCI was assessed using a validated test battery; global deficit scores (GDS) quantified overall performance. Biomarkers in cerebrospinal fluid (CSF) were quantified by immunoassay: neurofilament light (NFL), total Tau (tTau), phosphorylated Tau 181 (pTau181), amyloid beta (Aβ)42, and Aβ40. Factor analysis was used to reduce biomarker dimensionality. Participants were 256 virally suppressed PWH and 42 PWoH, 20.2% female, 17.1% Black, 7.1% Hispanic, 60.2% non-Hispanic White, and 15.6% other race/ethnicities, mean (SD) age 56.7 (6.45) years. Among PWH, the best regression model for CSF showed that higher tTau (*β* = 0.723, *p* = 3.79e-5) together with lower pTau181 (*β* = −0.510, *p* = 0.0236) best-predicted poor neurocognitive performance. In univariable analysis, only higher tTau was significantly correlated with poor neurocognitive performance (tTau *r* = 0.214, *p* = 0.0006; pTau181 *r* = 0.00248, *p* = 0.969). Among PWoH, no CSF biomarkers were significantly associated with worse NCI. Predicted residual error sum of squares (PRESS) analysis showed no evidence of overfitting. Poorer neurocognitive performance in aging PWH was associated with higher CSF tTau, a marker of age-related neuronal injury, but not with biomarkers of amyloid metabolism. The findings suggest that HIV might interact with age-related neurodegeneration to contribute to cognitive decline in PWH.

**Supplementary information:**

The online version contains supplementary material available at 10.1007/s13365-022-01081-4.

## Introduction

As people with HIV (PWH) age on virally suppressive antiretroviral therapy (ART), they become susceptible to age-related neurodegenerative disorders such as Alzheimer’s disease (AD). The biomarker phenotype of AD as compared to HIV-associated neurocognitive disorders (HAND) is distinct, supporting differences in the underlying pathophysiology. For example, one study reported that cerebrospinal fluid (CSF) total Tau (tTau) was increased in a group with AIDS dementia complex (ADC) compared to unimpaired PWH and younger PWoH, but lower than in an Alzheimer’s disease comparison group (Gisslen et al. 2009). There was no preferential accumulation of hyper-phosphorylated tau (pTau) as found in Alzheimer’s disease. A second study similarly found higher CSF tau levels in neurocognitively impaired PWH compared to unimpaired PWH (Cysique et al. 2015). A study of 94 PWH demonstrated a significant correlation between higher CSF tTau and worse HAND severity as measured by the Memorial Sloan-Kettering scale, HIV dementia scale, and Mosaic test [56] (Steinbrink et al. 2013). A comprehensive review of these and other papers (Brown et al. 2014) found that while not the case in all studies (Clifford et al. 2009), the preponderance found elevated CSF tTau in PWH with neurocognitive impairment. Proposed mechanisms of increased CSF tau in HAND include nitroxidative stress, altered proteostasis, DNA damage, chronic microglial activation triggered directly by viral replication or by HIV-viral proteins (e.g., glycoprotein 120, transactivator of transcription), and epigenetic dysregulation (Brown et al. 2014; Cho et al. 2017; Levine et al. 2016; Chemparthy et al. 2021).

Alterations in amyloid processing also are seen in HIV. Several studies reported increased accumulation of Aβ in autopsy brains of PWH compared to age-matched PWoH (Achim et al. 2009; Soontornniyomkij et al. 2012; Morgello et al. 2021). Research has delineated several mechanisms by which HIV infection might promote cerebral amyloid deposition. First, HIV proteins may influence AD biomarkers. For example, HIV Tat protein specifically induced both the release of the amyloidogenic Aβ42 peptide and the accumulation of cell-bound amyloid aggregates in primary rat fetal hippocampal cell cultures (Aksenov et al. 2010). Tat modulates APP processing through a variety of mechanisms, including inhibition of the Ab-cleaving enzyme, neprilysin (Rempel and Pulliam 2005). Additionally, the HIV nef and glycoprotein (gp) 120 proteins interact with Lamp2 and Beclin-1, interfering with autophagy, leading to Aβ and Tau accumulation (Fields et al. 2013). gp120 increased Aβ release from and transport across human brain microvascular endothelial cells (Liu et al. 2017). HIV gp120 enhanced Aβ accumulation in human brain microvascular endothelial cells through the Alpha7 nicotinic acetylcholine receptor (α7 nAChR). Amyloid precursor protein (APP) binds the HIV-1 Gag polyprotein, promoting secretase-dependent cleavage of APP, resulting in the overproduction of toxic Aβ isoforms (Chai et al. 2017). Finally, in simian immunodeficiency virus (SIV) + rhesus macaques and patients diagnosed with HIV, brain region–specific upregulation of APP and Aβ (40 and 42) was found in astrocytes (Sil et al. 2020); this was associated with find increased expression of β-site cleaving enzyme (BACE1), APP, and Aβ in human primary astrocytes exposed to HIV Tat.

Antiretroviral medications (ARVs) may contribute to altered APP processing. Thus, combination ARV treatment of SweAPP N2a cells increased Aβ generation and markedly inhibited microglial phagocytosis of FITC-Aβ1-42 peptides in murine microglia (Giunta et al. 2011). In another study, chronic exposure to tenofovir, a component of most current regimens contributed to Aβ accumulation as well as spatial learning and memory deficits in mice (Zulu et al. 2020).

Previous studies have evaluated biomarkers of age-related neurodegeneration in HIV such as amyloid PET, cerebrospinal fluid (CSF) Tau, and CSF Aβ42. Results are mixed, with some studies finding increases in PET amyloid ligand uptake (Ances et al. 2012), reductions in CSF Aβ42 indicative of higher brain amyloid deposits^5^ and increases in CSF Tau (Peterson et al. 2014) and neurofilament light (NFL) in PWH versus PWoH, while others do not (Clifford et al. 2009; Ortega and Ances 2014). CSF Aβ42 is either normal or diminished in PWH with HIV-associated neurocognitive disorders (HAND). Three studies found CSF Aβ42 to be reduced in PWH with HAND (Clifford et al. 2009; Krut et al. 2013; Brew et al. 2005). In contrast, three other studies have observed no changes in CSF Aβ42 in PWH relative to PWoH (Gisslen et al. 2009, 2016; Ances et al. 2012). These studies differed in the age, viral suppression, and antiretroviral exposure of their subjects and had relatively small sample sizes. Amyloid imaging using [11C] PiB has also not demonstrated increased extracellular amyloid fibrillar deposits in PWH with HAND (Steinbrink et al. 2013).

Here, we aimed to evaluate a more comprehensive panel of biomarkers of age-related neurodegeneration in CSF in relation to neurocognitive impairment in a larger cohort of older PWH than has been studied previously.

## Materials and methods

### Design and participants

This was a cross-sectional analysis of prospectively enrolled research participants recruited between October 1999 and March 2019 from community sources and clinics at the San Diego HIV Neurobehavioral Research Center (HNRC) and at academic centers in southern California participating in the California NeuroAIDS Tissue Network (CNTN). Inclusion criteria were PWH with viral suppression and PWoH; all underwent lumbar puncture. Exclusion criteria were active neurological illnesses other than HIV and active psychiatric or substance use disorders that might interfere with completing study evaluations. Individuals with non-HIV-related neurocognitive confounding conditions likely to explain neurocognitive impairment were excluded using a previously described method (Heaton et al. 2010). All participants signed informed consent documents approved by local IRBs.

### Clinical evaluations

Neurocognitive function was assessed using a comprehensive, standardized battery covering seven domains commonly affected by HIV (Heaton et al. 2010). Raw test scores were transformed into demographically adjusted *T*-scores based on normative samples of PWoH. *T*-scores for each test were converted into deficit scores that ranged from 0 (*T*-score ≥ 40, no impairment) to 5 (*T*-score < 20, severe impairment) and then averaged across tests to obtain a Global Deficit Score (GDS) (Carey et al. 2004). Neurocognitive impairment was defined as a GDS score ≥ 0.50 (Carey et al. 2004). A subset of participants (*N* = 247/256) who answered standardized questions on the impact of cognitive impairment on activities of daily living were classified with respect to the HIV-associated neurocognitive disorders (HAND) system (Grant et al. 2014): neurocognitively normal (NCN) asymptomatic neurocognitive impairment (ANI), mild neurocognitive disorder (MND), and HIV-associated dementia (HAD).

### Laboratory evaluations

HIV disease was diagnosed by enzyme-linked immunosorbent assay with Western blot confirmation. HIV RNA in plasma was measured using commercial assays and deemed undetectable at a lower limit of 50 copies/ml. CD4 T cells were measured by flow cytometry and nadir CD4 by self-report. CSF was collected in polypropylene tubes as previously described to minimize protein adsorption (Vanderstichele et al. 2016) and stored at −80 °C. Samples were thawed on the day of analysis, centrifuged at 14,000 × *g* for 10 min, and plated in Quanterix-supplied 96-well plates. The fully automated Simoa platform (www.quanterix.com) was used to measure biomarkers in CSF, including neurofilament light (NFL), total tau (tTau), phosphorylated Tau 181 (pTau181), amyloid beta (Aβ42), and Aβ40. For NFL, the assay used the Quanterix NFLight Advantage kit (#103,186) at the manufacturer’s recommended 1:100 dilution for CSF. Aβ42, Aβ40, and tTau (Watson et al. 2020) were assayed with the Quanterix Neuro 3-plex A multiplex kit (#101,995) at the recommended 1:80 dilution for CSF. CSF pTau181 was assayed with the Quanterix CSF-only kit (#103,377) at the recommended 1:4 dilution.

To minimize the number of freeze–thaw cycles, samples were analyzed in two Quanterix instruments simultaneously per matrix for the NFL and Neuro 3-plex assays immediately following the first thaw and then refrozen at −80 °C. The CSF samples were then re-thawed and analyzed for pTau181. Samples were batched to run in one plate (~80 samples) to reduce platform time at room temperature to < 3 h to minimize Aβ42, and Aβ40 loss (Rozga et al. 2019). All samples were analyzed in duplicate. The analysis of all samples within a matrix was performed in 5 separate runs within a week, using the same kit lot.

Each batch run of samples for all assays contained calibrators generating a standard curve, and controls from the same kit lot, described by the manufacturer. Assay performance was consistent with the manufacturer’s specifications. Inter-assay variability of NFL averaged < 8% percent coefficient of variation (%CV); for tTau, Aβ42, and Aβ40 the variability averaged < 7% CV, 4% CV, and 7%CV, respectively; and for pTau181 the variability averaged < 4% CV. The intra-assay variability of the duplicate measurements averaged < 20% CV (NFL < 5% CV, tTau < 5% CV, Aβ42 < 6% CV, Aβ40 < 8% CV, and pTau181 < 5% CV). Lower and upper limits of quantitation for CSF assays were: NFL 68.6, 40,000 pg/mL; tTau 8.8, 575 pg/mL; Aβ42 16.8, 3624 pg/mL; Aβ40 60, 9520 pg/mL; pTau181 4.8, 352 pg/mL. We also calculated ratios of Aβ42:Aβ40, tTau:Aβ42, and pTau:Aβ42.

### Statistical analyses

Participant characteristics were summarized using means, standard deviations, percentages, *t*-tests, and Wilcoxon tests, as appropriate. Biomarker values were log_10_-transformed to improve the normality of their distributions for parametric analyses. Univariable associations between GDS and biomarker levels were analyzed using Pearson *r* values. Interactions between HIV serostatus and biomarker levels were examined. Confounds known to influence the selected biomarkers or neurocognitive outcomes were evaluated using multivariable modeling, and included demographics. Multivariable associations used stepwise, mixed regression (p to enter/exit 0.1). To assess the possibility that the final selected model was overfit, we calculated the predicted residual error sum of squares (PRESS). Analyses were repeated while stratifying by HIV serostatus. Secondary analyses related the biomarkers and their ratios to potential confounds (demographics and medical comorbidities). Analyses were conducted using JMP Pro® version 15.0.0 (SAS Institute Inc., Cary, NC, 2018).

## Results

Participants were 256 virally suppressed PWH and 42 PWoH, 20.2% female, 17.1% black, 7.1% Hispanic, 60.2% non-Hispanic white, and 15.6% other race/ethnicities, mean (SD) age 56.7 (6.45) years. Among PWH, median (interquartile range [IQR]) current and nadir CD4 + T cells were 458 cells/μL (277, 677) and 57 cells/μL (11, 200), respectively; all took ART and were virally suppressed. PWH and PWoH were demographically similar (Table 1), but females were proportionately lower in PWH than PWoH (16.4% versus 42.9%). Global impairment rates were 46.1% for PWH and 16.7% for PWoH (*p* = 0.0002).

**Table 1:** Demographic and clinical characteristics of the study participants

	**All**	**PWoH**	**PWH**	***p*** (PWH vs. PWoH)
*N*	298	42	256	0.794
Age—years (mean ± SD)	56.7 ± 6.45	57.8 ± 6.04	56.5 ± 6.50	0.240
Sex female (*N*, %)	20.2%	18 (42.9%)	42 (16.4%)	2.74e-5
Education—years (mean ± SD)	13.6 ± 3.04	13.5 ± 2.10	13.6 ± 3.18	0.794
Race/ethnicity				
Non-Hispanic Black (*N*, %)	50 (17.1%)	18 (22.5%)	82 (16.4%)	0.654
Hispanic	21 (7.1%)	2 (5.0%)	19 (7.48%)	–
Non-Hispanic White	177 (60.2%)	21 (52.5%)	156 (61.4%)	–
Other	43 (15.6%)	8 (20%)	38 (14.8%)	–
Current CD4 (median, IQR)	–	–	458 (277, 677)	–
Nadir CD4 (median, IQR)	–	–	57 (11, 200)	–
HIV duration—years (median, IQR)	–	–	16.2 ± 7.68	–
On antiretroviral therapy (*N*, %)	–	–	256 (100%)	–
Undetectable plasma viral load on ART	–	–	256 (100%)	–
Diabetes mellitus (*N*, %)	52 (18.6%)	8 (19.1%)	44 (18.6%)	0.491
Hypertension (*N*, %)	203 (72.7%)	18 (42.9%)	159 (67.4%)	1.17e-12
Hyperlipidemia (*N*, %)	108 (3.88%)	11 (26.2%)	97 (41.1%)	0.0617
Hepatitis C virus seropositive (*N*, %)	53 (2.83%)	12 (28.6%)	41 (28.3%)	0.970

Table 2 shows median (IQR) values for each of the biomarkers and biomarker ratios in CSF for PWoH and PWH. PWH had higher values than PWoH for CSF NFL. Older age was associated with higher CSF NFL for PWH (*r* = 0.303, *p* = 8.82e-6), and showed a trend for PWoH (*r* = 0.164, *p* = 0.299). Older age correlated with higher CSF tTau for both PWH (*r* = 0.135, *p* = 0.0313) and PWoH (*r* = 0.425, *p* = 0.005). Older age significantly correlated with higher CSF pTau181 values in PWH (*r* = 0.207, *p* = 0.0009, and showed a trend for PWoH (*r* = 0.285, *p* = 0.0676. No demographics were related to CSF Ab42 and Ab40.

**Table 2:** CSF biomarker levels for PWoH and PWH and effect sizes for the differences between the two groups

Marker	PWoH	PWH	Effect size (Cohen’s *d*)
**NFL (log**_**10**_** pg/mL)**	**2.79 ± 0.211**	2.90 ± 0.28*	**0.0386**
**tTau (log**_**10**_** pg/mL)**	**1.96 ± 0.246**	**2.00 ± 0.309**	**0.0346**
pTau181 (log_10_ pg/mL)	1.61 ± 0.179	1.68 ± 0.238^	0.310
Aβ42 (log_10_ pg/mL)	2.95 ± 0.203	3.00 ± 0.271	0.252
Aβ40 (log_10_ pg/mL)	4.01 ± 0.154	4.04 ± 0.240	0.169
tTau:Aβ42	0.666 ± 0.084	0.656 ± 0.097	0.196
pTau181:Aβ42	0.547 ± 0.061	0.561 ± 0.08	0.0378

^*^ PWH vs. PWoH *p* < 0.05; ^ 0.05 < *p* $\le$ 0.10

**Fig. 1:**
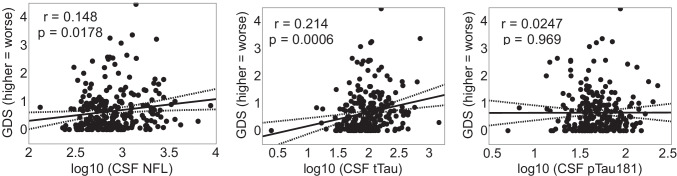
Scatterplots of univariable correlations of CSF NFL, tTau, and pTau181 with cognitive performance (GDS, global deficit score) in PWH

In PWH, a factor analysis including each of the 5 CSF biomarkers yielded 2 factors, with factor 1 loading on Ab40 and Ab42, and factor 2 loading on NFL, tTau, and pTau. Higher levels of factor 2 were associated with worse neurocognitive performance (*r* = 0.211, *p* = 0.0007), while factor 1 was not (*r* = − 0.0139, *p* = 0.826). Among the biomarkers comprising factor 2, both higher levels of tTau and NFL were each significantly correlated with worse neurocognitive performance (NFL *r* = 0.148, *p* = 0.0178; tTau *r* = 0.214, *p* = 0.0006;) while pTau was not (*r* = 0.00247, *p* = 0.969) (Fig. 1). Among PWoH, no CSF biomarkers or ratios were related to GDS (all ps > 0.20).

As shown in Table 3, the HAND group (*N* = 154; 59 ANI, 27 MND, 7 HAD) had higher CSF NFL and tTau values and CSF tTau:Aβ42 ratios than NP normal (ps 0.0354, 0.0109, and 0.0032). The three HAND subgroups had similar biomarker and ratio levels for all other biomarkers. PWH with HAND had higher values than those without, and the HAD subgroup had the highest values.

**Table 3:** Biomarker levels in PWH without neurocognitive impairment (NP normal), the combined HAND group, and the 3 HAND subgroups. All biomarker values are log_10_ transformed

	NP normal	All HAND	p	ANI	MND	HAD	p
*N*	93	154	–	59	27	7	–
**CSF NFL**	**2.85 ± 0.251**	**2.92 ± 0.287**	**0.0354**	2.93 ± 0.302	2.89 ± 0.251	2.96 ± 0.318	0.722
**CSF tTau**	**1.92 ± 0.299**	**2.02 ± 0.285**	**0.0109**	2.014 ± 0.28	2.02 ± 0.291	2.00 ± 0.354	0.980
CSF pTau	1.67 ± 0.219	1.68 ± 0.239	0.553	1.68 ± 0.246	1.674 ± 0.213	1.759 ± 0.296	0.688
CSF Aβ40	4.02 ± 0.190	4.04 ± 0.286	0.684	4.06 ± 0.154	3.97 ± 0.473	4.12 ± 0.155	0.315
CSF Aβ42	2.97 ± 0.249	3.02 ± 0.266	0.175	3.02 ± 0.215	3.01 ± 0.372	3.08 ± 0.191	0.814
**CSF tTau:Aβ42**	**0.677 ± 0.102**	**0.636 ± 0.090**	**0.0032**	0.666 ± 0.071	0.683 ± 0.138	0.648 ± 0.086	0.620
CSF pTau:β42	0.561 ± 0.068	0.561 ± 0.094	0.975	0.557 ± 0.079	0.567 ± 0.129	0.568 ± 0.061	0.882
CSF β40:β42	0.0932 ± 0.0627	0.0866 ± 0.0490	0.356	0.082 ± 0.027	0.116 ± 0.105	0.100 ± 0.047	0.0742

Supplementary Table 4 shows that, for all participants, each of the CSF biomarkers was strongly positively correlated with the others.

### Associations of demographics with neurocognitive performance and biomarkers

Among PWH, increasing age was not related to neurocognitive performance. Among PWH, increasing age correlated with higher CSF NFL (*r* = 0.303, *p* = 8.82e-7), CSF tTau (*r* = 0.1351, *p* = 0.0313), and CSF pTau181 (*r* = 0.207, *p* = 0.0009), but not CSF Aβ42 (*r* = 0.00961, *p* = 0.879) or CSF Aβ40 (*r* = 0.0660, *p* = 0.2950), but not the other biomarkers. Among PWH, sex was not significantly related to any of the CSF biomarkers (ps > 0.20). None of the biomarkers was significantly associated with ethnicity or education (ps > 0.05).

### Associations of medical comorbidities and antiretroviral therapy with neurocognitive performance and biomarkers

Among PWH, GDS was not related to any of the following common comorbidities: hypertension, diabetes, hyperlipidemia, cardiac disease, tobacco smoking, hepatitis C virus serostatus, chronic pulmonary disease, myocardial infarction, renal disease, congestive heart failure, or head injury. Biomarkers and biomarker ratios also did not relate to any of these comorbidities. All PWH took ART and were virally suppressed at the time of assessment. The median (IQR) total duration of exposure to ART was 108 (49, 167) months. Longer duration of ART exposure correlated with lower CSF Ab42 levels (*r* = −0.170, p = 0.0410), but was not related to the other biomarkers or biomarker factors. Duration of ART was not significantly related to GDS (*r* = 0.0790, *p* = 0.345). The most common regimen type taken by participants was a combination of a protease inhibitor (PI) with 2 nucleoside reverse transcriptase inhibitors (NRTIs), comprising 40.3% of the total. Additional regimen types, in order of frequency, were: a non-nucleoside NRTI plus 2 NRTIs (23.6%), a 3-class regimen of a PI an NNRTI and two NRTI (21.5%), an integrase inhibitor (II) with two NRTIs (9.87%), a PI plus an II (0.0858%), a PI with an NNRTI (0.0858%), and a regimen with a PI, an NNRTI, an II and 2 NRTIs 3.00%).

### Multivariable analysis of CSF biomarker relationships to neurocognitive performance

Among PWH, the best regression model showed that higher tTau (*β* = 0.723, *p* = 3.79e-5) together with lower pTau181 (*β* = −0.510, *p* = 0.0236) best-predicted poor neurocognitive performance. In univariable analysis, only higher tTau was significantly correlated with poor neurocognitive performance (tTau *r* = 0.214, *p* = 0.0006; pTau181 *r* = 0.00248, *p* = 0.969). Age was not significant in an adjusted model (*p* = 0.293). To evaluate goodness of fit and possible overfitting, we compared the predictive *R*^2^ (0.0401) to the model *R*^2^ (0.0267). The similar values indicate no evidence of overfitting.

In PWoH, the multivariable model found that no CSF biomarkers were significantly related to neurocognitive performance. In a multivariable model, the interaction of HIV serostatus with CSF tTau was not significant (*p* = 0.225). Together, both HIV serostatus and CSF tTau were significant (ps 0.00128 and 0.00048, respectively; model *p* = 1.33e-5).

## Discussion

We found strong, statistically significant associations of neurocognitive impairment with biomarkers of age-related axonal injury (total Tau protein) in CSF in virally suppressed, cognitively unconfounded PWH, but not in PWoH. No correlations were found with biomarkers of amyloid metabolism, though a caveat here is that the number of PWoH was relatively small, limiting statistical power. These results highlight the emerging role of age-related neuronal injury in the aging population of PWH. As anticipated based on prior studies, we found higher levels of CSF NFL, a marker of axonal injury, correlated with poorer neurocognitive performance in PWH.

With the rapidly expanding intersection of long-term HIV and age-related neurodegenerative disorders, the field is increasingly interested in characterizing how, and to what extent, neurodegenerative disorders might contribute to the persisting high prevalence of neurocognitive impairment in HIV (Heaton et al. 2010). Older PWH have a higher risk of impairment than younger PWH. Our findings suggest that age-related neurodegeneration might amplify cognitive impairment related to HIV and its treatment. There is likely a subset of PWH with neurodegeneration that is advanced enough to put them on the tipping point between normal age-related and AD-related degeneration. We have previously estimated that 30–50% of older PWH have a memory impairment profile that is more similar to AD than to HAND, as previously described (Sundermann et al. 2021).

### Tau protein in CSF

Tau is implicated in the assembly and stability of microtubules, while phospho-tau, its hyper-phosphorylated form, is produced in the course of inflammation and detaches from microtubules, forming neurofibrillary tangles (Calcagno et al. 2016). CSF Tau protein elevations are not disease-specific, occurring in multiple tauopathies including progressive supranuclear palsy and AD (Wagshal et al. 2015). We found a correlation between worse neurocognitive performance and higher levels of tTau in CSF among PWH. This is similar to some previous reports (Cysique et al. 2015; Clifford et al. 2009; Brew et al. 2005; Green et al. 2000), but different from others (Clifford et al. 2009). In agreement with some previous reports (Steinbrink et al. 2013; Clifford et al. 2009), though not all (Cysique et al. 2015; Brew et al. 2005; Ozturk et al. 2019), we found that CSF pTau181 by itself was not increased in relation to cognitive impairment.

### Amyloid beta in CSF

In AD, amyloid aggregates in the extracellular space of the brain parenchyma and its soluble forms are sequestered away from CSF (Joachim et al. 1988). Comparing results in different studies is problematic due to differences in neurocognitive impairment severity, HIV disease duration, patient age, diagnostic criteria, and/or treatment status. Additionally, several preanalytical variables can interfere with the results. In particular, Aβ42 levels are affected by the time of sample collection, the type of tube used to collect and store the CSF, and the number of freeze–thaw cycles. We took care to avoid these errors. In agreement with other studies (Clifford et al. 2009; Peterson et al. 2014; Fields et al. 2020), we found that worse neurocognitive performance was not associated with CSF Aβ42 or Aβ40 levels in PWH.

#### CSF NFL

Our finding that higher CSF NFL concentrations correlated with worse neurocognitive function is in agreement with the great preponderance of previous reports among PWH. Increased CSF NFL is a nonspecific marker of axonal damage in multiple neurological conditions, including AD, stroke, head trauma, and multiple sclerosis (Khalil et al. 2018).

Strengths of this study include the large, diverse cohort of virally suppressed PWH, enhancing generalizability, the inclusion of an uninfected comparison group, the comprehensive neurocognitive testing, the careful exclusion of neurocognitive confounding conditions, and a factor analysis designed to control false discovery. Additional analyses showed no evidence of overfitting in the statistical models described here.

Limitations of this study include the indirect relationships of CSF to brain markers, the study’s cross-sectional nature, and the inability to ascertain causality. A recently described, newer assay used antibodies with higher specificity for Aβ42 and 40 (Thijssen et al. 2021), but these antibodies were not available when we conducted the study. The kit that we used contained antibodies that detect intact peptides as well as the N-terminal fragments (Aβx-42 and Aβx-40). It will be valuable to perform future studies with the fragment-specific antibodies and compare the findings. We did not characterize ApoE genotype, a known risk factor for cognitive decline and amyloidosis in older individuals (Hsiung et al. 2004; Reas et al. 2019; Liu et al. 2013), but our previous work demonstrated that ApoE4 genotype did not increase the risk of HAND (Morgan et al. 2013). While it is plausible that underlying mechanisms of neurodegeneration explain the observed associations, a third unobserved variable, such as neuroinflammation, might generate these correlations. Indeed, inflammation is correlated with axonal injury and increased NFL (Romme Christensen et al. 2013; Villar et al. 2015). Finally, our study was underpowered to evaluate the associations of biomarkers with neurocognitive impairment in PWoH.

If the observed alterations in biomarkers are causally related to neurocognitive impairment in PWH, then interventions might be designed to stop or reverse these alterations. In vitro or animal model work may provide preliminary answers to this question.

## Supplementary information

Below is the link to the electronic supplementary material.

Supplementary file1 (DOCX 13 KB)

## Data Availability

Data will be made available upon request.
