# Supplementary material for: Higher cerebrospinal fluid biomarkers of neuronal injury in HIV-associated neurocognitive impairment
Source: J Neurovirol. 2022 Jun 8;28(3):438–45. doi: 10.1007/s13365-022-01081-4 (PMC9470698; doi:10.1007/s13365-022-01081-4)
Supplement: Supplementary file 1 — Supplementary file1 (DOCX 13 KB) [file 13365_2022_1081_MOESM1_ESM.docx]

**Supplementary Table 4** CSF biomarker intercorrelations

| CSF | log_10_ TTau | | log_10_ P-Tau181 | | log_10_ Aβ42 | | log_10_ Aβ40 | |
| --- | --- | --- | --- | --- | --- | --- | --- | --- |
|  | r | p | r | p | r | p | r | p |
| log_10_ pTau181 | 0.543 | 2.95e-24 |  |  |  |  |  |  |
| log_10_ Aβ42 | 0.514 | 1.62e-21 | 0.478 | 2.03e-18 |  |  |  |  |
| log_10_ Aβ40 | 0.524 | 1.98e-22 | 0.527 | 1.09e-22 | 0.894 | 5.4e-105 |  |  |
| log_10_ NFL | 0.459 | 6.42e-17 | 0.344 | 1.06e-9 | 0.209 | 0.000277 | 0.223 | 0.0001 |
